# Theranostics in Interventional Oncology: Versatile Carriers for Diagnosis and Targeted Image-Guided Minimally Invasive Procedures

**DOI:** 10.3389/fphar.2019.00450

**Published:** 2019-05-09

**Authors:** Nils Degrauwe, Arnaud Hocquelet, Antonia Digklia, Niklaus Schaefer, Alban Denys, Rafael Duran

**Affiliations:** ^1^Department of Diagnostic and Interventional Radiology, Lausanne University Hospital and University of Lausanne, Lausanne, Switzerland; ^2^Department of Oncology, Lausanne University Hospital and University of Lausanne, Lausanne, Switzerland; ^3^Department of Nuclear Medicine and Molecular Imaging, Lausanne University Hospital and University of Lausanne, Lausanne, Switzerland

**Keywords:** transarterial chemoembolization, radioembolization, SIRT, HCC, theranostics, ablation, cancer, interventional oncology

## Abstract

We are continuously progressing in our understanding of cancer and other diseases and learned how they can be heterogeneous among patients. Therefore, there is an increasing need for accurate characterization of diseases at the molecular level. In parallel, medical imaging and image-guided therapies are rapidly developing fields with new interventions and procedures entering constantly in clinical practice. Theranostics, a relatively new branch of medicine, refers to procedures combining diagnosis and treatment, often based on patient and disease-specific features or molecular markers. Interventional oncology which is at the convergence point of diagnosis and treatment employs several methods related to theranostics to provide minimally invasive procedures tailored to the patient characteristics. The aim is to develop more personalized procedures able to identify cancer cells, selectively reach and treat them, and to assess drug delivery and uptake in real-time in order to perform adjustments in the treatment being delivered based on obtained procedure feedback and ultimately predict response. Here, we review several interventional oncology procedures referring to the field of theranostics, and describe innovative methods that are under development as well as future directions in the field.

## Introduction

In the past decades, combined efforts have improved our understanding of various diseases at the molecular level, in particular in the field of cancer (Hanahan and Weinberg, 2011). For instance, we learned that malignancies are heterogeneous entities containing cells with different genetic and epigenetic landscapes and that primary tumors and associated distant metastases may also dramatically differ from a molecular point of view (Dagogo-Jack and Shaw, 2018). Thus, one type of treatment may be effective only in a subset of patients with the same disease or may target only a certain fraction of cancer cells within a same patient (Easwaran et al., 2014). As a consequence, numerous targeted therapies have been developed and are now a cornerstone in the management of various malignancies, alone or in combination with other treatment such as chemotherapy, radiation therapy, interventional radiology or surgery (Sawyers, 2004; Adam and Kenny, 2015).

To address this increasing need for patient-tailored therapies, technological advances have been made in targeted diagnostics, for example using specific radionuclides or nanoparticles (Chen et al., 2014). Given disease heterogeneity among and within cancer patients, it is essential to evaluate the presence of a target to predict whether or not a patient may benefit from therapy. Molecular imaging may also assist for accurate distinction between healthy tissue and tumor, helping surgeons and interventional radiologists to completely eradicate malignancies without affecting surrounding tissue or organs, thereby improving outcomes while reducing morbidity (Willmann et al., 2008). This combined necessity for targeted diagnostics and therapeutics led to the emergence of a new field, named “theranostics,” which refers to methods that couples a therapy or intervention with simultaneous diagnostic information for a specific target (Jo et al., 2016). This may have the advantages of providing information about the presence and biodistribution of a target, predicting off-target effects, aiding in determination of optimal dosage, and monitoring response to treatment (Lee and Li, 2011). The use of radioiodine for the treatment of thyroid cancer is a classical and emblematic example of the early days of theranostics which was developed back in the 1940s, iodine-131 being a γ- and β-emitter and thus beneficial for both imaging and treatment, respectively (Silberstein, 2012). Later on, fluorescent markers, imaging probes and nuclear imaging agents were progressively developed and sometimes coupled with therapeutic agents, in order to gain insight into molecular pathways and therapeutic efficacy while simultaneously treating cancer lesions (Kelkar and Reineke, 2011). More recently, complex nanoparticle-based theranostic platforms with both diagnostic and therapeutic capabilities have been developed to specifically detect, image and target tumor lesions (Janib et al., 2010; Xie et al., 2011).

Interventional oncology is a rapidly expanding field of interventional radiology. Interventional oncology is based on sophisticated image-guided treatments and procedures, and therefore constitutes a pioneering specialty in the field of minimally invasive and precision medicine, allowing simultaneous diagnosis, therapy, and real-time monitoring of treatment efficacy (Adam and Kenny, 2015). Through using the most cutting-edge technologies to better see, selectively target and treat cancer, interventional oncology is playing an ever-increasing role in the management of cancer patients. In this review we detail various theranostic methods applied to interventional oncology as well as future directions in the field.

## Catheter-Based Intra-Arterial Therapies

### Transarterial Chemoembolization

Transarterial chemoembolization (TACE) is one of the first theranostic methods that was used in the field of interventional radiology, combining diagnosis of hepatic tumors with tumor “tagging,” simultaneous tumor treatment and real-time treatment evaluation (Iwai et al., 1984; Konno et al., 1984;Ohishi et al., 1985; Yumoto et al., 1985). Since the first developments, TACE has been widely utilized in the treatment of hepatocellular carcinoma (HCC) and is currently the standard of care for patients with unresectable, intermediate-stage disease according to the Barcelona Clinic Liver Classification (BCLC). It is also performed in other indications for HCC patients such as downstage to surgery, early stage or less frequently advanced-stage disease (European Association for the Study of the Liver, 2018; Marrero et al., 2018). Moreover, TACE has been increasingly performed in patients with metastatic liver disease (Mahnken et al., 2013).

TACE exploits the fact that normal liver cells are preferentially vascularized by portal venous flow while cancer cells almost exclusively depend on arterial blood supply. This dual vascularization of the liver offers a unique therapeutical opportunity to specifically target liver tumors. From a femoral access, a microcatheter is super-selectively positioned, under fluoroscopic guidance, into the relevant branch(es) of the hepatic artery supplying the tumor(s). Injection of iodine-based soluble contrast medium is used to delineate the arterial anatomy and demonstrate areas of tumor blush to allow for accurate microcatheter positioning into the tumor feeding vessels, avoiding as much as possible non-target embolization of non-tumoral liver parenchyma. Once the injection site is selected, the “chemo”-“embolization” payload (i.e., chemotherapy together with an embolizing agent) is infused allowing to reach a high drug concentration that could otherwise not be achieved by a systemic delivery, thus maximizing anticancer drug efficacy while minimizing systemic toxicity (Duran et al., 2015).

#### Conventional TACE

**FIGURE 1:**
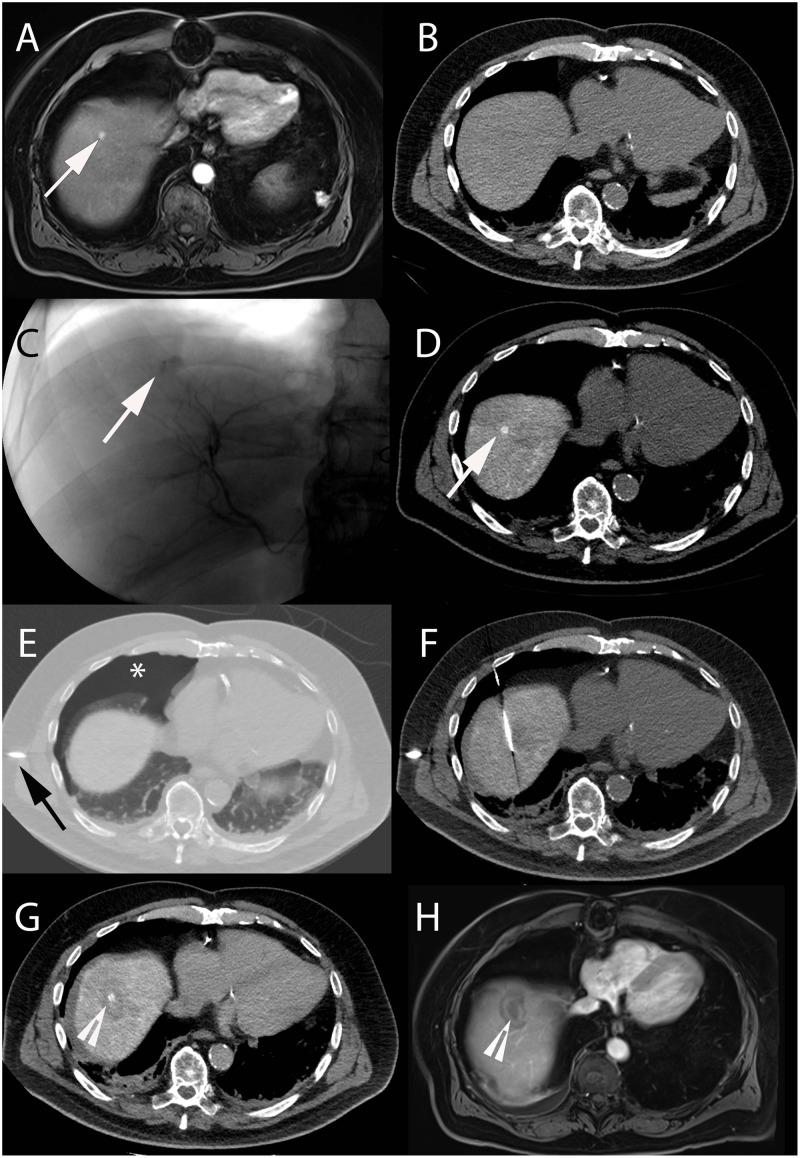
Intra-arterial hepatic administration of Lipiodol for tumor marking and visualization before radiofrequency ablation in a 64-year-old male with a history of alcoholic cirrhosis and HCC. The tumor lesion (arrow) easily identified on contrast-enhanced arterial phase T1-weighted MRI **(A)** was neither visible on ultrasound nor on unenhanced **(B)** and contrast-enhanced (not shown) CT images. **(C)** Selective angiography from the right hepatic artery demonstrated an area of contrast blush corresponding to the HCC lesion (arrow). **(D)** Unenhanced CT-scan performed immediately after Lipiodol injection from the right hepatic artery showed avid Lipiodol uptake by the tumor that became visible on CT (arrow). **(E)** Iatrogenic CO2 pneumothorax (^∗^) was performed using a Veress needle (black arrow) to allow lesion targeting without touching the lung. **(F)** The radiofrequency probe could then be precisely placed into the lesion using CT-guidance. **(G)** Post-ablation unenhanced CT showed hypodense area surrounding the tumor with satisfactory ablation margins (arrowhead). **(H)** Contrast-enhanced arterial phase T1-weighted MRI performed 1-month after treatment showed complete tumor ablation (arrowhead).

The most frequently prescribed TACE is called conventional TACE (cTACE). cTACE consists of the administration of an emulsion composed of an anticancer agent (e.g., doxorubicin, the most frequently used cytotoxic agent) mixed with Lipiodol (Guerbet, Paris, France), a poppy seed oil-based chemical (Lencioni et al., 2016). Besides this drug carrier ability, Lipiodol combines other unique, theranostic properties. It is radiopaque and thus enables precise real-time monitoring of the emulsion delivery during treatment. Moreover, Lipiodol has the unique ability to be selectively up-taken and long-term retained by HCC and other hyperarterialyzed liver tumors (Konno et al., 1984; Yumoto et al., 1985). Thus, besides its role in cTACE, Lipiodol can be injected alone in the hepatic artery as a tumor seeking agent allowing for a precise cartography of liver tumors. A tumor that is not visible on ultrasound or CT can be thus “marked” and becomes visible on x-ray imaging for subsequent targeting, e.g., biopsy and/or thermal ablation (Figure 1). Furthermore, cTACE also capitalizes on the moderate embolic effect of Lipiodol, related to its oily/viscous nature, which allows for arterial and even portal embolization resulting in tumor hypoxia and cell death (Nakamura et al., 1988). The emulsion is slowly injected until the whole treatment is administered or vascular stasis is obtained. This is followed by embolization of the tumor feeding vessels, using gelatin sponge (Gelfoam) or microparticules, which further occludes the tumor vasculature increasing tumor ischemia and preventing rapid washout of the emulsion, thus maximizing the exposure of the cytotoxic agent with the tumor cells.

Extensive work has been performed to investigate crucial tumor parameters {such as abnormal tumor vasculature [size, density, distance from cancer cells, increased vascular permeability and tumor retention of macromolecules [caused by poorly functional lymphatic vessels], tumor and extracellular matrix [size, histopathology, microenvironment, necrosis, acidity, hypoxia, etc.,] reviewed in (Tredan et al., 2007)} and physiochemical drug and Lipiodol, as well as emulsion characteristics that influence tumor uptake and locoregional drug delivery [reviewed in (Idee and Guiu, 2013)]. In particular, physiochemical properties of Lipiodol-cytotoxic drug emulsion have been the focus of research efforts to develop the optimal emulsion (water-in-oil vs. oil-in-water), which should ideally deposit specifically in the tumors, have the ability to deliver high on-target drug dose while plasmatic concentrations remain low, and be stable for slow drug release and prolonged exposure in tumor tissue. Early works demonstrated that water-in-oil emulsions had higher embolic effect and drug carrier properties with longer drug release time as opposed to oil-in-water emulsion (de Baere et al., 1995; Kan et al., 1997). A recent pre-clinical study compared the use of a classical emulsion (oil/water ratio: 1/1) vs. an emulsion with a higher oil/water ratio (3/1) combined with an emulsifier. The latter showed increased theranostic properties, including higher accumulation in tumors compared to adjacent healthy liver, higher intra-tumoral and lower systemic concentrations of doxorubicin (Deschamps et al., 2017a). During emulsion preparation, continuous and incremental injections of doxorubicin in Lipiodol resulted in almost 100% formation of water-in-oil type emulsion, and had increased stability as compared to bolus injections (Deschamps et al., 2017b). Moreover, the use of specific biodegradable nanoparticles, that adsorb at the water/oil interface, demonstrated to improve therapeutic properties of an oxaliplatin-Lipiodol emulsion, including prolonged chemotherapy releasing time and decreased plasmatic peaks (Deschamps et al., 2018). Further research is needed to find the best parameters to formulate stable water-in-oil emulsions and increase reproducibility. Indeed, considerable heterogeneity exists in the emulsion characteristics used in cTACE. Of particular importance is a thorough *in vivo* investigation of the behavior of well characterized *in vitro* emulsions.

#### Drug-Eluting Beads TACE

In addition to cTACE, drug-eluting beads may be used as well for TACE, better known as drug-eluting beads TACE (DEB-TACE). In DEB-TACE, microparticules (also commonly referred to as “beads”) are loaded with an anticancer drug, suspended in iodinated soluble contrast medium and infused into target tumor tissues. DEBs provide a more reproducible platform and standardized approach when compared to cTACE for which many drugs and emulsion preparations are utilized with no consensus or universally adopted regimen (Lencioni et al., 2012). Many embolic microparticulate platforms have been successfully tested and are summarized in (Giunchedi et al., 2013; Fuchs et al., 2017). DEB-TACE allows for precise drug delivery to the tumor with decreased systemic toxicity (Varela et al., 2007; Lammer et al., 2010; Lewis and Dreher, 2012). Once trapped into intra-tumoral as well as tumor feeding vessels at the tumor periphery, the anticancer agent is eluted into the surrounding tissues. Locoregional anticancer efficacy is thus achieved by the synergistic combination of targeted deposition of the beads into tumor tissue reaching high drug concentration together with the embolic effect of the beads themselves. Indeed, embolization not only prevents rapid drug washout but constitutes the main trigger of cancer cell death (Brown et al., 2016). Newer DEBs platforms use a smaller microparticle size as *in vitro* testing and preclinical models demonstrated mechanistic advantages over larger bead size. Although, drug penetration seems to be relatively independent of the microparticule size, smaller beads penetrate deeper into targeted tissues achieving better spatial resolution and density when compared to larger beads size, potentially achieving a better tumor drug coverage (Dreher et al., 2012; Caine et al., 2018).

**FIGURE 2:**
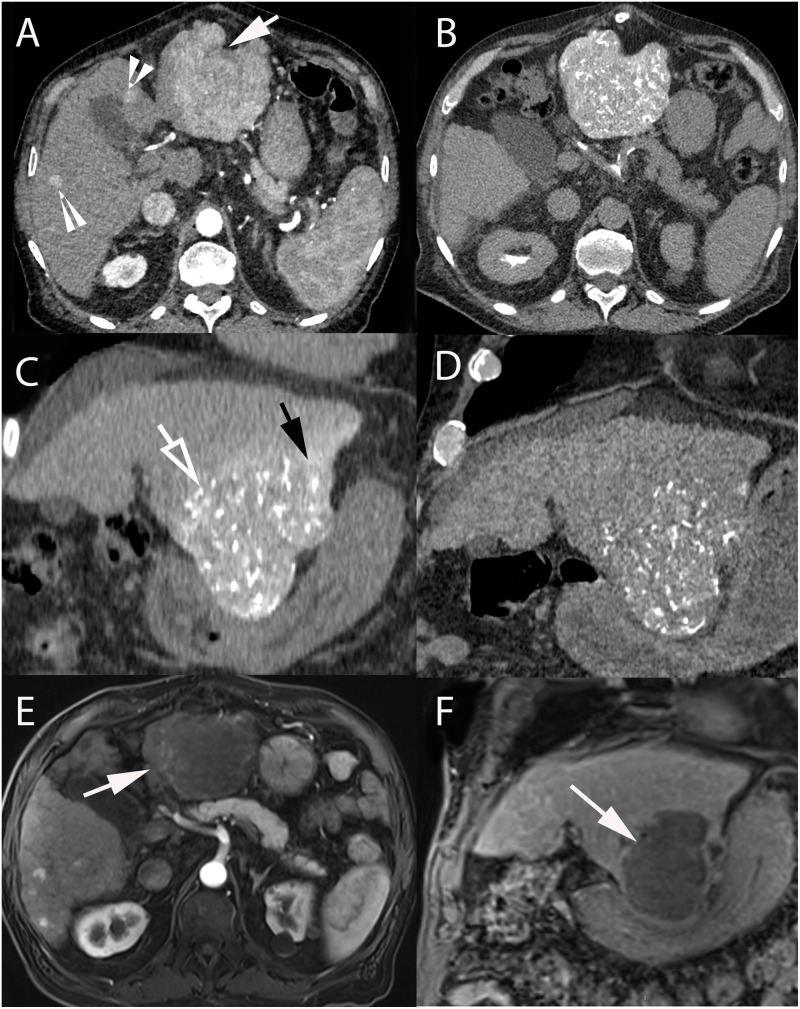
74-year-old male with hepatitis C cirrhosis and multifocal HCC treated with radiopaque drug-eluting beads loaded with doxorubicin. **(A)** Contrast-enhanced CT-scan (arterial phase) showing a large HCC in segments II-III (arrow) and small HCC lesions in segments IV and V (arrowheads). Axial **(B)** and coronal **(C)** unenhanced CT after selective administration in the left hepatic artery clearly demonstrating two types of attenuation: from radio-opaque drug eluting-beads (DC Bead LUMI loaded with doxorubicin) deposited into the tumor (hollow arrow) and soluble contrast medium used during catheterization and embolization (black arrow). **(D)** Coronal unenhanced CT image at 1-month post TACE showing that radiopaque beads deposition was still readily visible while the soluble contrast medium had long “washed out.” Axial (arterial phase) **(E)** and coronal (portal phase) **(F)** contrast-enhanced T1-weighted MRI performed at 1-month after 2 consecutive TACEs demonstrated extensive necrosis of the treated lesion (white arrow).

**FIGURE 3:**
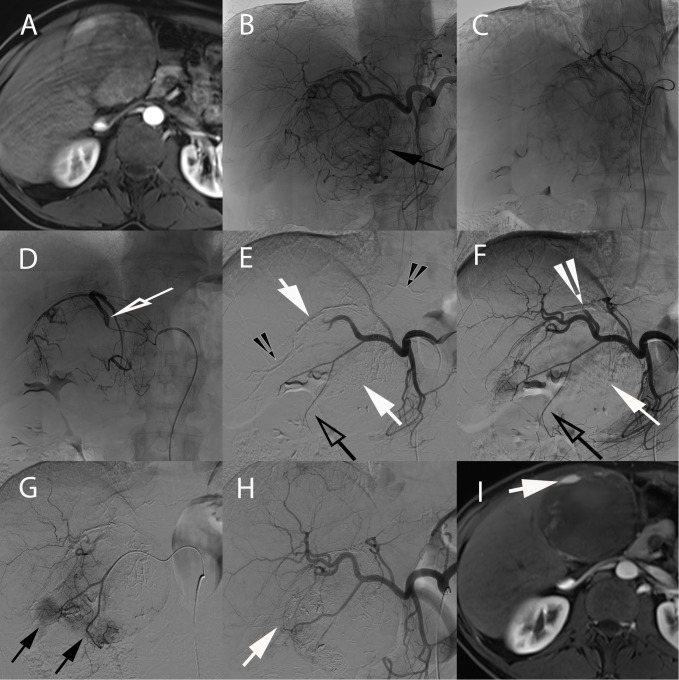
58-year-old male with hepatitis B and C cirrhosis and voluminous HCC of the left liver treated with radiopaque drug-eluting beads allowing real-time monitoring of treatment deposition. **(A)** Axial contrast-enhanced (arterial phase) T1-weighted MR image showing voluminous enhancing lesion of the left liver corresponding to an HCC. **(B)** Celiac angiography depicting large area of contrast blush corresponding to the tumor (black arrow). Superselective angiography with the microcatheter positioned in the left hepatic artery **(C)** and in the hypertrophied artery of segment IV (hollow arrow) **(D)**, both supplying the tumor. Injection of radio-opaque drug eluting-beads (DC Bead LUMI loaded with doxorubicin) was performed from these 2 arteries. **(E)** Early and **(F)** delayed phases of same celiac angiography. **(E)** Delivered beads are readily visible, both within the tumor and peritumoral vessels (white arrows) and in non-target regions (black arrowheads). **(F)** The tumor blush is not visible anymore and complete blow stagnation is visible in segment IV artery (white arrowhead). However, no beads are seen at the bottom and lateral portion of the tumor [**(E,F)**, black hollow arrows]. **(G)** Superselective microcatheterization and angiography from the cystic artery, showing areas of tumor blush consistent with residual untreated regions (black arrows). **(H)** Completion celiac angiography demonstrates treatment deposition in previously untreated tumor portion (arrow) and no residual tumor blush. **(I)** Axial contrast-enhanced (arterial phase) T1-weighted MR image performed 1-month after treatment showing large area of necrosis with minimal residual enhancing area in the anterior part of the tumor (arrow).

As opposed to cTACE, DEB-TACE lacks Lipiodol and may not provide adequate feedback of treatment deposition into the tissue. Indeed, the soluble contrast medium used to suspend the beads allows visualization of the treatment to monitor real-time delivery into targeted tissues and prevent non-target embolization. Once the beads are delivered, presence or absence of soluble contrast retention into targeted tumor tissues can be used as surrogate markers of treatment location when using intraprocedural imaging such as cone-beam computed tomography (CBCT) or multidetector CT (Golowa et al., 2012; Wang et al., 2013). However, these signs are ephemeral due to contrast washout and the actual bead location is unknown. As a result, novel imageable, radiopaque beads have been developed to better visualize treatment delivery and identify non-target embolization to modify the procedure in real-time (Duran et al., 2016; Tacher et al., 2016; Figure 2). Moreover, precise intra-procedural assessment of radiopaque beads location may help identify tumor regions at risk of being untreated either on projection images (Figure 3) or CBCT (Levy et al., 2016).

#### Multimodality Lesion Visualization, Targeting, and Treatment Assessment

**FIGURE 4:**
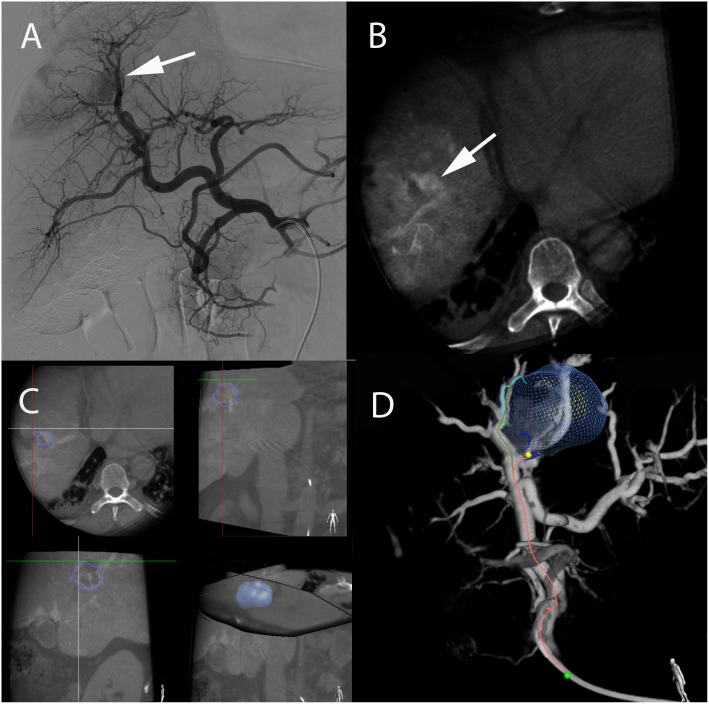
Real-time tumor tracking and detection of HCC feeder in a 57-year-old male with hepatitis B and alcoholic cirrhosis. **(A)** Angiography from the celiac axis showing an area of blush corresponding to the target tumor (arrow) and multiple vessel superposition of putative tumor-feeding arteries. This complex arterial anatomy made tumor feeders very difficult to identify. **(B)** Intraprocedural contrast-enhanced CBCT performed with soluble contrast medium injection through the microcatheter in the proper hepatic artery depicted a nodular hyperattenuating area corresponding to the tumor (white arrow). **(C)** Based on that CBCT, the tumor could be precisely segmented in 3D and a 3D reconstruction of the arterial anatomy of the liver could be obtained. **(D)** A tumor-feeding vessels detection software (EmboGuide, Philips) could then be used which displayed the target lesion together with 3D reconstructed angiogram. The microcatheter tip (green dot), tumor feeding vessel (in red) and its branches (green, light and dark blue) are shown. This superimposed 3D target tumor with tumor feeders can be superimposed to real-time fluoroscopy (not shown) allowing for optimal superselective microcatheter placement before treatment administration.

Regardless of the type of TACE that is chosen, the most cutting-edge technologies are used to better see, reach and treat liver tumors, and assess/predict treatment response. The advent of CBCT in the angiosuite has changed the landscape of procedures performed in interventional radiology, in particular of catheter-based intra-arterial therapies. The C-arm system used to perform fluoroscopy and two-dimensional (2D) projection images can now be utilized to generate 3D volumetric CT images (Tacher et al., 2015). Traditionally, 2D projection images of digital subtraction angiography (DSA) are used to identify tumor “blushes” and evaluate hepatic arteries. However, identification of tumors with DSA is hazardous as it depends on tumor vascularity and size, with hypervascular and/or big lesions being more easily identifiable as opposed to hypovascular and/or small lesions. With CBCT, tumors are displayed with an image quality comparable to conventional CT/MR images with the ability to identify tumors that are not visible on angiography (Miyayama et al., 2009, 2013; Schernthaner et al., 2015, 2016). Moreover, DSA depicts super-imposed arteries leading to potential misinterpretation of tumor feeding vessels and administration of the treatment into a wrong location. After a single injection of soluble contrast medium into the proper hepatic artery, 3D arterial anatomy of the whole liver can be obtained with CBCT allowing an accurate assessment of the vasculature. Thus, the use of 3D imaging in the angiosuite has led not only to the better visualization of primary and secondary liver tumors and hepatic arteries during treatment but also to the identification of new lesions leading to real-time treatment modification (Miyayama et al., 2009, 2013; Schernthaner et al., 2015, 2016). Co-registration of the 3D volumetric CT dataset obtained with CBCT with fluoroscopy may help catheterization, tumor feeding vessels identification and targeting (Figure 4; Deschamps et al., 2010; Iwazawa et al., 2013; Miyayama et al., 2013). Moreover, previous 3D cross-sectional imaging (CT, MRI, and PET-CT) can be co-registered with the CBCT images to further help tumor targeting (Abi-Jaoudeh et al., 2012).

Early assessment of treatment success following TACE is crucial as incomplete treatment favors recurrence and negatively impacts survival. This is particularly true for cancer patients in whom the clinical course is highly dependent on disease progression in the liver such as HCC. Tumor response is typically assessed on MRI (or CT) at 4–6 weeks after the procedure. However, tumor response prediction obtained intra-procedurally has the advantage of helping to modify the course of treatment in real-time and guide future treatment decisions. The theranostic properties of Lipiodol are ideal to predict response. Lipiodol uptake by HCCs during TACE correlates with various clinical response criteria and tumor necrosis. On follow-up imaging, a lack of residual contrast enhancement, high spontaneous attenuation and diffuse distribution of Lipiodol within the lesion are all surrogate markers of tumor response (Monsky et al., 2010; Riaz et al., 2010). Studies correlating imaging and pathology also suggest that the response to cTACE is related to the lesion hypervascularity and size of the tumor feeding artery, being 3–5 times superior if its diameter is ≥ 0.9 mm (Kwan et al., 2012). More importantly, a strong correlation was found between the degree of Lipiodol deposition into HCC lesions as assessed quantitatively on intraprocedural CBCT and tumor response at 1-month on MRI, highlighting the per-procedural response prediction of Lipiodol (Wang et al., 2015). Similar intraprocedural tumor response prediction using CBCT was also shown for DEB-TACE with a significant correlation between the decrease in tumor enhancement on CBCT immediately after DEB-TACE and complete and/or partial tumor response at 1-month follow-up MRI (Loffroy et al., 2013). In a preclinical model of liver cancer (rabbit VX2), the volume of drug-eluting radiopaque beads loaded with the antiangiogenic vandetanib quantified on CBCT was a significant prognostic factor of tumor necrosis (Duran et al., 2018).

### Catheter-Based Intra-Arterial Delivery of Theranostics Nanoparticles

Theranostic nanomedicine is an emerging field in biomedicine which is expected to play an increasing role in patient tailored management. In cancer therapy, nanoplatforms have the dual benefit of being specific imaging probes able to detect and diagnose tumor lesions and at the same time drug carriers preferentially delivering a specific therapeutical agent to those lesions (Xie et al., 2011). However, despite recent improvements, theranostics nanoparticles are still facing challenges such as toxicity and low selective targeting efficiency when delivered systemically (Janib et al., 2010). Thus strategies to maximize nanoparticles delivery is critical. A way to achieve adequate tumor targeting relies on catheter-based intra-arterial delivery of theranostics nanoparticles. This strategy has the advantage of delivering high doses of nanoparticles locoregionally, potentially overcoming problems related to biodistribution and tumor uptake. In a rabbit model for liver cancer, hollow gold nanoparticles [(64)Cu-labeled PEG-HAuNS] were mixed with Lipiodol and intra-arterially delivered in the hepatic artery with significantly greater tumor uptake when compared to systemic delivery of the nanoplatform. Imaging properties of the nanoparticles allowed for subsequent treatment monitoring using PET-CT (Tian et al., 2013). Other theranostic nanoplatforms such as hydroxyapatite nanoparticles (used as vector for gene therapy) (Li et al., 2012), doxorubicin loaded in superparamagnetic iron oxide (SPIO) nanoparticles (Lee et al., 2013; Mouli et al., 2013) or porous magnetic nano-clusters (Dox-pMNCs) (Jeon et al., 2016), mixed with Lipiodol were successfully used for the intra-arterial treatment of liver cancer. Moreover, the magnetic susceptibility of the latter two enables precise MRI monitoring and quantification of the treatment. An alternative strategy is to use doxorubicin-loaded SPIO nanoparticles which are further linked together with polyvinyl alcohol to form a composite for TACE (Liang et al., 2017). Taken together these promising results highlight the potential nanotheranostic platforms to overcome challenges of systemic administration and achieve meaningful results in locoregional delivery once translated in the clinic.

### Selective Internal Radiation Therapy

**FIGURE 5:**
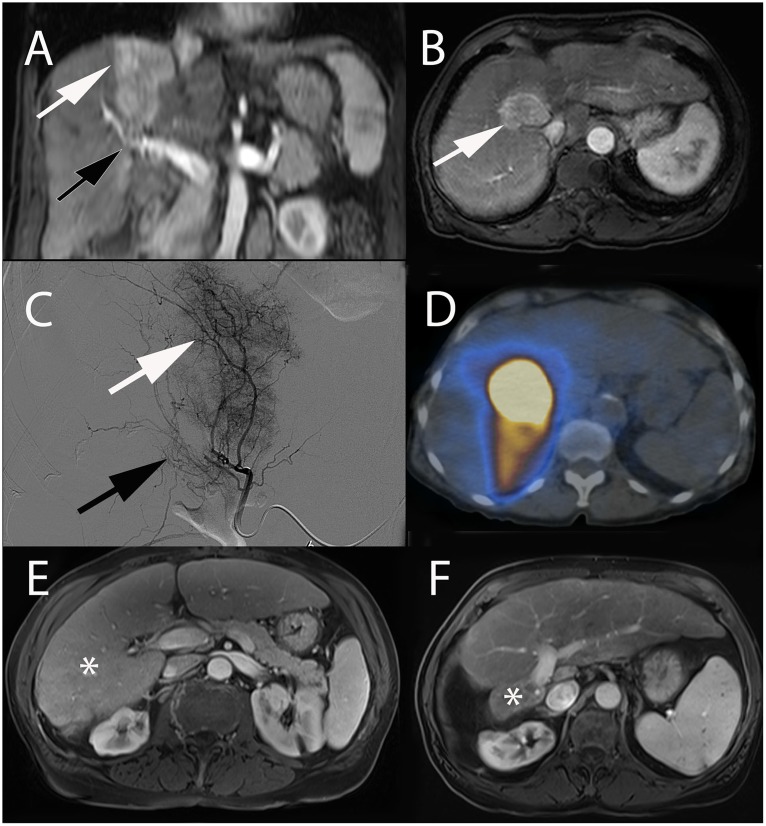
Radiation ablative extended right hepatic lobectomy with SIRT in a 65-year-old female patient with hepatitis C cirrhosis and HCC. Coronal **(A)** and axial **(B)** contrast-enhanced (arterial phase) T1-weighted MR images showing large area of enhancement corresponding to HCC centered in segment VIII and IV (white arrow). Note the tumoral portal vein invasion (black arrow). **(C)** Selective segment VIII angiography demonstrated a large area of contrast blush corresponding to the tumor (white arrow) and portal vein invasion (black arrow). **(D)** SPECT/CT fused axial image acquired after administration of ^90^Y-microspheres showing activity mostly confined to segment VIII. Axial contrast-enhanced (portal venous phase) T1-weighted MR image obtained before **(E)** and after **(F)** two consecutive SIRT. Note post-treatment radiation ablative extended right hepatic lobectomy (^∗^ = segment IV-VIII), with marked compensatory left liver hypertrophy.

Selective internal radiation therapy (SIRT), also called radioembolization, is primarily used for the treatment of HCC. Patients with intermediate-stage disease (BCLC B), who are not suitable for TACE or progressed after TACE, have large tumors and macrovascular invasion of the portal vein are considered good candidates (Sangro et al., 2012; Duran et al., 2015; Garin et al., 2015). Moreover, the use of SIRT is particularly interesting for HCC patients who are surgical candidates, in particular for those with a small future liver remnant volume or those listed for transplant (Vouche et al., 2013; Theysohn et al., 2014; Salem et al., 2016). SIRT has the ability, not only of treating the targeted tumor, but also of increasing the non-treated liver volume (Vouche et al., 2013; Theysohn et al., 2014). Moreover, when the disease can be super-/selectively targeted, ablative radiation doses can be administered achieving radiation segmentectomy, hepatectomy or lobectomy (Figure 5; Vouche et al., 2013). Also, in patients with limited liver disease who are not candidates for surgery or thermal ablation, SIRT can be an alternative therapy using ablative radiation doses. Furthermore, SIRT has been increasingly prescribed in patients with unresectable cholangiocarcinoma and secondary liver cancers with promising results (Bridgewater et al., 2014; Van Cutsem et al., 2014; Pavel et al., 2016).

SIRT shares technical similarities with TACE given that it also takes advantage of the preferential arterial supply of liver tumors. Selective arterial catheterization is performed under fluoroscopic control to find the optimal injection site to treat the tumor(s), avoiding as much as possible unnecessary treatment of nontumoral liver and thus maximizing treatment/radiation concentration to malignant cells. Radioembolization is based on the administration of radioactive compounds, such as 131-iodine-labeled Lipiodol or microspheres containing yttrium-90 (^90^Y), the latter being the more widely used isotope for the treatment of liver cancers (Raoul et al., 1997; Salem et al., 2013). ^90^Y is a pure β-emitter with minor tissue penetration (mean of 2.5 mm), thus the patient does not need to be isolated for radioprotection and can be discharged the same day of the procedure. Within 2 weeks after treatment, the majority of radiation (>95%) is delivered into the targeted tissues (Sangro et al., 2012). As opposed to TACE, the main anticancer effect is caused by the radiation itself while embolization is only a minor contributor (Sato et al., 2006). Two types of microspheres are currently available for ^90^Y-radioembolization, made of glass (TheraSphere, Biocompatibles UK Ltd., Farnham, United Kingdom) and resin (SIR-Spheres; Sirtex Medical Ltd, Australia). Despite different physico-mechanical and activity properties, both systems have shown similar efficacy in clinical trials (Sangro et al., 2012; Van Der Gucht et al., 2017).

#### Patient-Tailored Simulation Angiogram and Therapy

**FIGURE 6:**
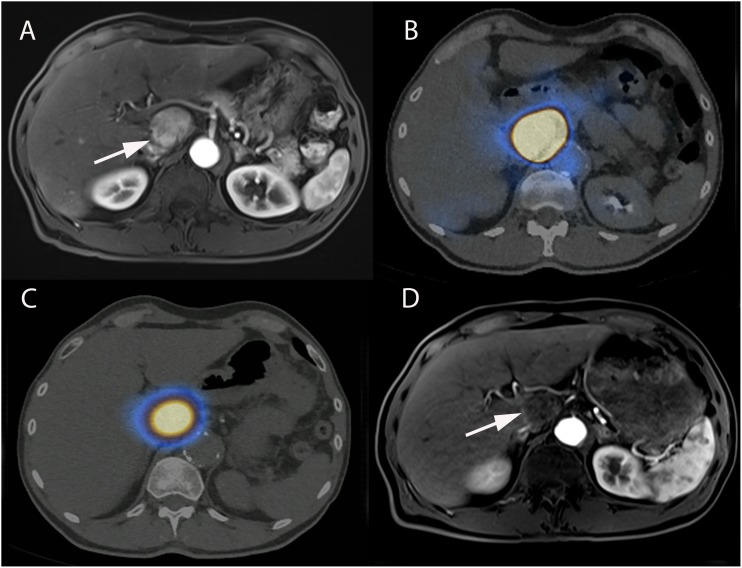
Super-selective SIRT of caudate lobe in a 63-year-old male patient with alcoholic cirrhosis and HCC. **(A)** Axial contrast-enhanced (arterial phase) T1-weighted MR image showing enhancing tumor in caudate lobe corresponding to HCC (arrow). **(B)** Pre-treatment SPECT/CT realized after superselective administration of ^99m^Tc-MAA showing activity restricted to caudate lobe. **(C)** Post-treatment SPECT/CT performed immediately following ^90^Y-microspheres administration, showing excellent superselective tumor targeting and overlap with pretreatment ^99m^Tc-MAA. **(D)** Axial contrast-enhanced (arterial phase) T1-weighted MR image performed 3-months after SIRT showing a complete response with total disappearance of any intratumoral enhancement (arrow).

Before administration of the treatment itself, a diagnostic angiography is performed to evaluate liver and tumor vascular anatomy (Denys et al., 2015). Once the injection site is selected, a treatment simulation (“scout dose”) is performed with the infusion of technetium-99m-macroaggregated albumin (^99m^Tc-MAA). ^99m^Tc-MAA particles have approximately the same size as ^90^Y-microparticules and their distribution within the liver is expected to be roughly similar. Following ^99m^Tc-MAA administration, patients are imaged with single-photon emission CT (SPECT) to evaluate for potential extrahepatic and hepatopulmonary shunting, and to estimate the dose to the tumor and adjacent liver. This pre-procedural step is essential for treatment tailoring to the individual characteristics of each patient and for performing personalized predictive dosimetry (Figure 6; Kao et al., 2012; Garin et al., 2016). If favorable tumor to liver distribution is achieved and no significant extra-hepatic deposition is seen on ^99m^Tc-MAA SPECT, the radioembolization itself is usually conducted within 1-month or in some centers on the same day in a single-session (Gates et al., 2014; Denys et al., 2015).

Accurate treatment prediction is essential for patient safety and optimal personalized dosimetry. Extrahepatic shunting can lead to serious adverse effects such as radiation pneumonitis or gastrointestinal ulcers. At the moment, evaluation of the likelihood of extrahepatic shunting is based on ^99m^Tc-MAA distribution following intra-arterial administration. However it remains unclear whether there is a precise overlap between the pre-treatment ^99m^Tc-MAA-SPECT and the treatment itself, particularly because of differences in particle composition, shape, size and pharmacokinetics. Moreover, other factors such as microcatheter positioning, tumor and liver vasculature, blood flow, tumor histopathology and burden are likely to influence microparticules distribution. Indeed, ^99m^Tc-MAA remains a surrogate marker for ^90^Y-microspheres distribution and several studies reported that significant hepatopulmonary shunting occurs in > 10% of therapeutic interventions despite adequate pre-treatment ^99m^Tc-MAA analysis (Leung et al., 1995; Wondergem et al., 2013; Garin et al., 2016). Although the predictive ability of ^99m^Tc-MAA for ^90^Y-microspheres distribution is not perfect and has been debated, the pre-treatment dosimetry based on ^99m^Tc-MAA-SPECT was demonstrated to be accurate and predictive of response and survival in patients with different liver cancers (Flamen et al., 2008; Garin et al., 2012).

To palliate inherent differences between ^99m^Tc-MAA and ^90^Y-microspheres, efforts were conducted to develop microparticles that are similar to the radioactive microspheres used for treatment and provide better imaging capabilities. Several platforms are currently under investigation including sphere shaped ^99m^Tc-MAA (ROTOP Pharmaka, Germany) or positron-emitting microspheres such as starch-based microparticles radiolabeled by gallium-68 (^68^Ga; β^+^ emitter) or rhenium-188 (^188^Re: β^-^ and γ-emitter) (Verger et al., 2016) and the more clinically advanced holmium-166 (^166^Ho)-poly(L-lactic acid) microspheres (Zielhuis et al., 2006). The advantage of ^166^Ho-microspheres is that they can be used both as a scout dose for treatment planning and for the therapeutic dose itself, thereby overcoming the issues related to the different properties between ^99m^Tc-MAA and ^90^Y-microspheres. Moreover, ^99m^Tc-MAA overestimates the absorbed dose to the lungs compared to ^166^Ho-microspheres which may lead to patients receiving reduced treatment dose or for whom SIRT is eventually not performed (Elschot et al., 2014). In addition, paramagnetic properties of ^166^Ho can be used to assess the intrahepatic microspheres distribution and perform MRI-based dosimetry with good correlation with SPECT (γ-emitting properties of ^166^Ho allow for nuclear imaging) (Van De Maat et al., 2013). Moreover, preliminary evidence has demonstrated that ^166^Ho-radioembolization was safe with promising efficacy in patients with unresectable, chemorefractory liver metastases (Smits et al., 2012; Prince et al., 2018).

More recently, in an effort to provide a theranostic solution for discrepancies between treatment planning and the procedure itself, a method has been described that could create a direct link between the administration of modified MAA and the delivery of therapy (Spa et al., 2018). Adamantine functionalized MAA (Ad-MAA) (“guest”) were generated and either intravenously or locally injected into the spleen of mice followed by intravenous administration of a radiolabeled reactive macromolecule made of Cy 5 and Beta-cyclodextrin containing PIBMA polymers [99mTc-Cy5(0.5)CD10PIBMA39] (“host”). Guest and host moieties drive multivalent interactions, greatly increasing the binding affinity. The study showed approximatively a 10-fold increase in accumulation of host in the organs where MAA-Ad was injected/deposited, and thus functioning as a reactive pair (Spa et al., 2018). Taken together, this study showed that a pre-targeting approach could react with a multimeric host molecule that could be modified into a therapeutic agent for more specific and “single step” radioisotope delivery. In other words, this suggests that scout-scan distributions could closely mirror therapeutic radioisotope distributions.

#### Multimodality Lesion Visualization, Targeting, and Treatment Assessment

Similarly, to TACE, CBCT can be used to improve treatment planning and accuracy of SIRT. Intraprocedural CBCT can identify non-target embolization and/or regions of tumor that are not going to be treated, that DSA is unable to depict. This additional information prompts real-time treatment modifications such as microcatheter repositioning to improve treatment delivery and optimize safety and efficacy (Louie et al., 2009). Following radioisotope-microspheres delivery, patients are systematically imaged by SPECT to assess treatment distribution and potential non-target administration. Before this post-procedure assessment, an ideal scenario would be to have real-time treatment evaluation during SIRT, since post-treatment imaging/dosimetry cannot directly impact treatment delivery (as microspheres were already administered). One solution could also be to use MRI for real-time evaluation of treatment delivery using microspheres containing paramagnetic elements such as previously mentioned ^166^Ho (Van De Maat et al., 2013) or SPIO nanocrystals incorporated on glass microspheres which have shown accurate quantification of macroscopic intrahepatic distribution during SIRT in preclinical models (Gupta et al., 2008; Li W. et al., 2016). MRI-guided treatments come, however, with their own set of challenges such as lack of MR-compatibility of commonly used material in fluoroscopy such guidewires and microcatheters, availability and costs (Bock and Wacker, 2008). Another strategy could be to develop hybrid fluoroscopic and nuclear imaging modalities able to acquire real-time anatomical and nuclear images providing valuable intra-procedural feedback (Beijst et al., 2016).

### Other Locoregional Theranostic Radionuclide Therapies

Peptide receptor radionuclide therapy (PRRT) is a molecularly targeted treatment which involves the systemic administration of a radiolabeled peptide designed to selectively target receptors that are overexpressed on cancer cells (Bodei et al., 2013). The use of PRRT is particularly appealing for patients with metastatic gastroenteropancreatic neuroendocrine tumors (GEP-NETs). GEP-NETs usually have the molecular characteristic to overexpress somatostatin receptors, which can be targeted with radiolabeled somatostatin analogs such as [lutetium-177 DOTA^0^,Tyr^3^]octreotate (^177^Lu-DOTATATE) or [^90^Y-DOTA^0^, Tyr^3^]octreotide (^90^Y-DOTATOC). For example, ^177^Lu-DOTATATE has been increasingly used in the treatment of GEP-NETs with good results in terms of tumor response and overall survival (Brabander et al., 2017). However, complete responses remain rare and may be due to insufficient tumor uptake of the radiopeptides. As a result, many radioisotope pharmaceuticals originally conceptualized for systemic use are now being increasingly revisited for locoregional delivery. Indeed, selective intra-arterial administration of radiolabeled peptides is particularly interesting for the treatment of cancer patients with liver only or liver predominant disease, although systemic effects are also obtained after intra-arterial locoregional delivery. In particular, higher tumor absorbed radiation dose leading to better tumor response rates were obtained after intra-arterial delivery when compared to systemic administration as summarized in a recent review of seminal works (Feuerecker et al., 2017). Moreover, intra-arterial delivery could decrease the absorbed kidney dose, which constitutes PRRT’s main limitation (Pool et al., 2014). Taken together, these findings, although preliminary, highlight the fact that in addition to specific targeting of tumors with radiopeptides, increased therapeutic efficacy and specificity may be obtained by selective intra-arterial delivery. Further studies are needed to confirm these promising results gathered by locoregional intra-arterial delivery of radiolabeled peptides to challenge the actual hegemony of systemic PRRT.

## Image Guided Biopsies and Percutaneous Interventions

Resolution, accuracy and techniques available in the field of diagnostic radiology are constantly improving and progressing. We detect more and more lesions, often small in size, which may be ambiguous in nature and require confrontation to histopathology. Also, modern patient management, in particular cancer patient, requires a personalized approach with treatments tailored to molecular and histogenetic profiling of tumors. This may represent a challenge for interventional radiologists, e.g., liver biopsies of small tumors easily identifiable with MRI, but challenging to localize with ultrasound and/or CT which are typically used for procedural guidance. As a result, there is an increasing need for methods allowing to confirm proper needle positioning during biopsies or percutaneous interventions such as thermal ablations. This necessity to reach complex lesions for diagnostic and therapeutic purposes has led to the development of navigation systems and multimodality imaging techniques to facilitate the specific targeting of tissues of interest (Abi-Jaoudeh et al., 2012).

### Patient Tailored Therapies Using Advanced Guidance and Multimodality Imaging Techniques

**FIGURE 7:**
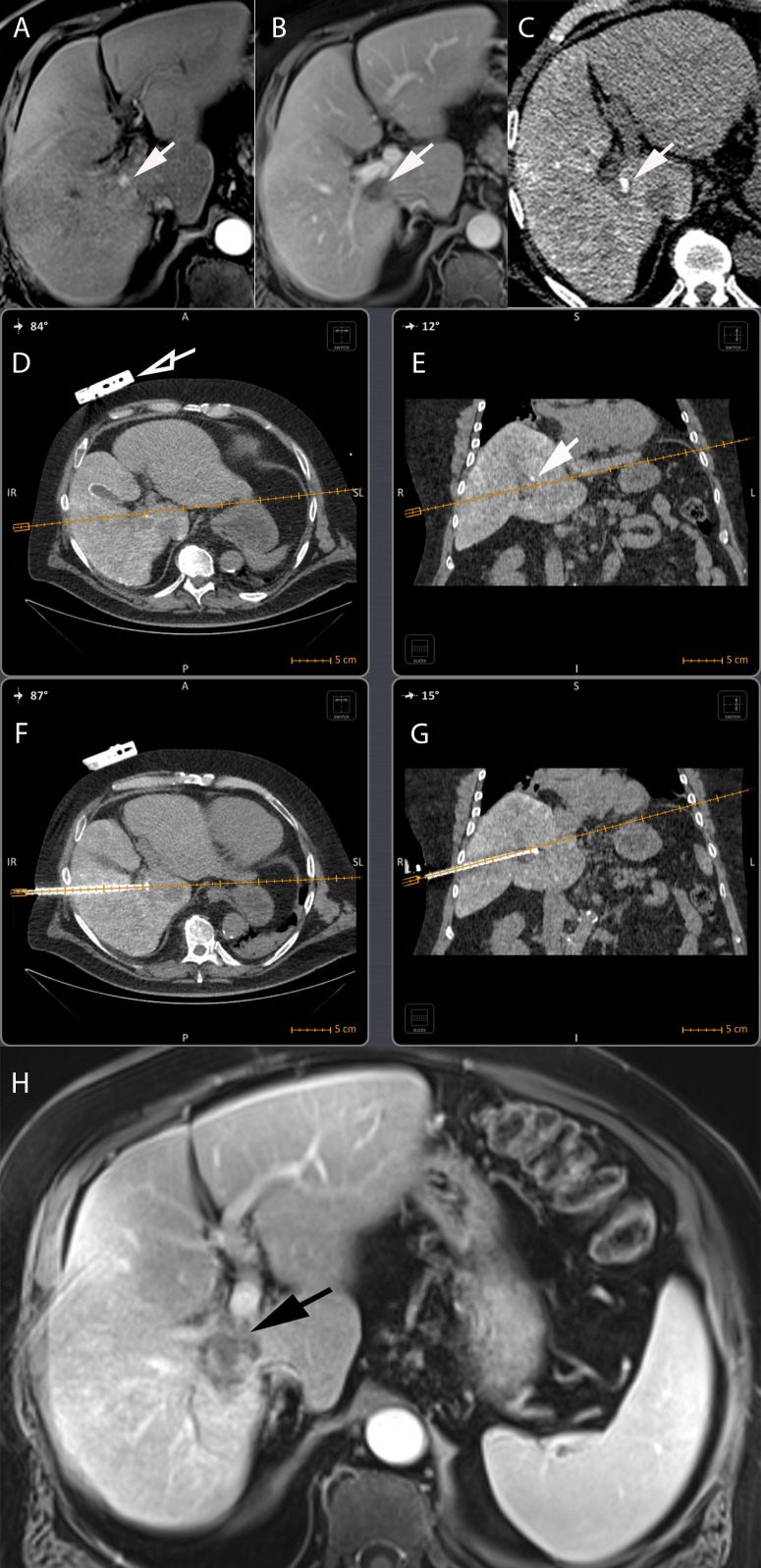
Electromagnetic CT navigation system for precise per-procedural guidance in a 67-year-old male with alcoholic cirrhosis and recurrent HCC following RFA. **(A)** Axial contrast-enhanced (arterial phase) T1-weighted MR image showing nodular enhancement adjacent to an ablation scare consistent with HCC recurrence following RFA (arrow). **(B)** Axial contrast-enhanced (portal venous phase) T1-weighted MR image showing tumor washout of the lesion, typical hallmark of HCC (arrow). **(C)** The lesion was neither visible on ultrasound nor unenhanced/contrast-enhanced CT. An intra-arterial hepatic administration of Lipiodol was thus performed to “tag” the tumor which became visible on unenhanced CT (arrow). The navigation system displays the needle path in real time in any plane wanted, here on axial **(D)** and coronal **(E)** views of a CT fused with the electromagnetic CT navigation system (Imactis SAS, La Tronche, France). The planned needle tract can thus be seen before the RFA probe is inserted in the patient. The fiducial of the navigation system is visible on the patient skin in **(D)** (hollow arrow). The tumor containing the Lipiodol is visible in **(E)** (arrow). Axial **(F)** and coronal **(G)** oblique views showing out-of-plane needle placement directly into the target lesion avoiding critical structures such as right portal vein and hepatic duct. **(H)** Axial contrast-enhanced (arterial phase) T1-weighted MR image showing complete ablation with no residual suspect area of enhancement (black arrow).

To overcome some technically challenging procedures, guidance systems can be used to help the interventional radiologist improve lesion targeting. This is particularly helpful for lesions that are small in size (Stattaus et al., 2007), may not be adequately visible on procedural imaging, are transiently apparent (e.g., hepatic lesion enhancing only on the arterial phase) or invisible, are located in anatomical regions difficult to reach or next to vulnerable structures. Many guidance systems have been tested such as electromagnetic, optical, laser and robotic navigations tools (Chehab et al., 2015). In particular, electromagnetic tracking and multimodality image fusion techniques are increasingly used to better see and reach targeted lesions. Previously available images, such as MRI, CT or PET-CT images, precisely depicting the targeted lesion, can be referenced, co-registered, and fused, with the procedural imaging for improved visibility and targeting. An electromagnetically tracked needle can be precisely positioned with spatial accuracy (Krucker et al., 2007, 2011). This allows the interventional radiologist to continuously monitor the targeted lesion and device placement (e.g., biopsy needle, ablation probe or microcatheter) using high-quality pre-procedural diagnostics and metabolic imaging, with a direct impact on procedures outcomes (Figure 7; Wallace et al., 2009; Braak et al., 2010; Krucker et al., 2011; Venkatesan et al., 2011; Ewertsen et al., 2013). Hence, integrated navigation platforms will certainly continue to gain in importance in the future, and may affine guidance toward particular lesions, expressing e.g., a particular biomarker identified by previous PET imaging using a specific radionuclide and may enable procedures that are not feasible with standard, single-modality image guidance.

In addition, new techniques such as optical molecular imaging are under development and consist of administration of fluorochromes for procedural and treatment guidance (Pysz et al., 2010). Fluorescence requires an emission light (typically provided by a laser) and a camera to detect the emission from the fluorophore. The ideal agent should have high contrast and sensitivity and thus favorable noise to signal ratio, give molecular information about the target, be safe, non-toxic, cheap and easy to use (Alander et al., 2012). Indocyanine green (ICG), probably the most frequently used fluorochrome, accumulates within or in close vicinity of HCC and liver colorectal metastases, with a favorable target-to-background ratio (Ishizawa et al., 2009; Kokudo and Ishizawa, 2012). Proof-of-concept of the potential use of ICG-guided percutaneous interventions was first demonstrated in pre-clinical models with development of an optical endoscope containing a laser and a camera, fitting coaxially into the sheath of a biopsy needle. After intravenous administration of ICG, samples obtained from the tumors showed a clear and sharp demarcation between the tumor and normal tissue, including in the biopsy core (Sheth et al., 2014). This was later translated to a prospective clinical trial highlighting the potential of molecular imaging in the field of minimally invasive procedures (Sheth et al., 2015). Many fluorescently labeled molecular markers are under development and will certainly enter routine clinical practice in the field of interventional oncology, improving diagnostic accuracy, reducing complications or sampling errors and helping to demarcate malignant from healthy tissue to improve outcomes of percutaneous interventions. In the future, one can imagine the development of new optical probes labeling specific targets *in vivo*, e.g., associated with an aggressive phenotype or poor outcome, which would allow better tumor sampling and/or for which the use of targeted therapy could be beneficial. Optimal molecular imaging could also be beneficial for *in vivo* assessment of tumor margin ablation during thermal ablation procedures, which can be critical as outlined in the next paragraph. Indeed residual fluorescence detected at the margin of an ablation could indicate residual disease.

### Thermal Ablation Therapies

Thermal-based ablations have been successfully used for the treatment of solid tumors in many locations such as liver, kidneys, adrenal glands, prostate, lungs, soft tissues, thyroid and bone. Ever-improving technology of ablative devices and their minimally invasive nature with greatly reduced morbidity when compared to surgery, together with their proven anticancer efficacy, have led to an exponential use of this technique (Bridgewater et al., 2014; Van Cutsem et al., 2014; Escudier et al., 2016; European Association for the Study of the Liver, 2018; Marrero et al., 2018). This is particularly the case for patients with HCC for whom thermal ablations, such as radiofrequency ablation (RFA), are routinely used as first-line therapy in very-early (BCLC stage 0; tumors < 2 cm diameter), and early stage disease as an alternative to surgery (BCLC stage A; up to three lesions ≤ 3 cm) (European Association for the Study of the Liver, 2018; Marrero et al., 2018; Vogel et al., 2018).

Thermal ablations may be heat-based [e.g., RFA, microwave ablation (MWA)] or cold-based (cryoablation). During ablation, a probe is directly inserted in the tumor under image guidance followed by heating or freezing beyond a cytotoxic threshold. The most commonly used technique is RFA in which a probe is connected to a radiofrequency current generator and inserted into a tumor, producing displacement of ions around the needle tip, heating and eventually leading to coagulation necrosis. MWA is based on the generation of electromagnetic waves inducing direct hyperthermic injury similarly to RFA. One advantage of MWA over RFA is a lower susceptibility to heat-sink effect, caused by close proximity of large vessels to the tumor, because MW depends less on thermal conduction and tissue can heat up several centimeters away from the needle (Ahmed et al., 2011). In addition, several antennas of MW can be used at the same time, acting synergistically and allowing treatment of larger and multifocal lesions simultaneously (Lubner et al., 2010). Despite these mechanistic advantages, MWA has not yet been demonstrated to be superior to RFA in the treatment of solid tumors such as HCC (Vietti Violi et al., 2018). Cryoablation uses freezing to lethal temperatures followed by thawing to cause extensive tumor necrosis.

#### Multimodality Lesion Visualization, Targeting, and Ablation Assessment

Despite being widely accepted in the medical community for the treatment of small tumors (≤3 cm), one of the key limitations for the expansion of ablations to larger tumors has been the difficulty in obtaining complete ablation of tumor margins (Livraghi et al., 2000). Thus, for all thermal ablation methods it is essential to have pre- and per-procedural visualization of the target lesion to adequately position the ablation probe and evaluate in real-time the ablation coverage to prevent early recurrence. Per-procedural guidance and monitoring of thermal ablation procedures are usually based on ultrasound or CT, although, as described above, navigation and multimodality fusion tools can be employed for challenging lesions (Wallace et al., 2009; Braak et al., 2010; Krucker et al., 2011; Venkatesan et al., 2011; Ewertsen et al., 2013). Ultrasound is a commonly used method for guidance of thermal ablation because it provides real-time information about needle and tumor(s) locations and, to some extent, the coverage of the ablation, although several studies reported discrepant results (Cha et al., 2000; Leyendecker et al., 2002). Indeed, during RFA (and MWA), artifacts appear on real-time ultrasound because of gas bubble formation during tissue heating. This may result in uncertainty in the ablation margins, which may be overestimated on ultrasound when compared to the actual tissue necrosis on histology (Leyendecker et al., 2002). Additionally, image quality may be hindered by tumor localization, poor acoustic window and coarse parenchymal echogenicity, particularly in cirrhotic liver. Better visualization of tumors can be achieved using microbubble-based contrast-enhanced ultrasonography, leading to a decreased in the number of sessions required to achieve complete tumor ablation (Masuzaki et al., 2011). Additionally, evaluation of treatment coverage can also be completed using immediate post-procedural contrast-enhanced ultrasonography with improved outcomes (Meloni et al., 2012; Wu et al., 2014).

Planning and per-procedural monitoring of thermal ablative treatment can be performed using CT-scan, in addition to or as an alternative method to ultrasonography (Park B.J. et al., 2009). An advantage of CT is the ability to precisely visualize critical structures close to the region being treated, allowing for better treatment planning or for the use of preventive measures, such as hydrodissection, to preserve non-target tissues. On unenhanced-CT, RFA treated regions appear as hypoattenuating regions, which are usually readily visible after ablation. However, the use of contrast-enhanced CT allows for better evaluation of the treated volume and is also useful for follow-up imaging. Indeed, an area of irregular peripheral enhancement may suggest recurrence, although discrimination with enhancing inflammatory reaction with granulation tissue surrounding necrosis can be challenging. Therefore, if available, the preferred method for follow-up and for evaluating recurrence after thermal ablative methods is MRI, which proved to be superior to contrast-enhanced CT (Dromain et al., 2002; Granata et al., 2013). Less frequently, because of costs and low availability, MR-guided ablations are performed in a small number of centers (Clasen et al., 2011). Theoretically, sensitivity of several MRI sequences to changes in temperature may serve to quantify and monitor ongoing thermal ablation (McDannold and Jolesz, 2000). PET-CT can also be used for both per-procedural guidance and immediate assessment of treatment success. Using a split-dose technique, a small amount of fluorine-18-fluorodeoxyglucose (^18^F-FDG) can be initially injected for lesion identification and procedural guidance. Because of its short half-life, ^18^F-FDG is no more or only marginally detectable at completion of the ablation. Thus, a second ^18^F-FDG dose can be administrated to identify potential residual viable tumor, allowing for immediate retreatment, if needed (Ryan et al., 2013). Moreover, immediate post-ablation PET-CT can accurately predict treatment success and recurrence, and proved to be superior to immediate post-procedural contrast-enhanced CT (Cornelis et al., 2016).

#### Combinatorial Approaches With Ablative Therapies

Besides technical improvements in lesion visualization, targeting and assessment of the treated area, strategies have been developed to improve the completeness of the ablation zone (regardless of the tumor size) and to expand the indication of thermal ablations to larger tumors (i.e., > 3cm). An approach consists of combining RFA with TACE. This strategy proved to be beneficial for the treatment of lesions above the 3 cm threshold for HCC patients and is routinely used in many centers. Indeed, two meta-analyses of randomized control trials comparing RFA vs. RFA+TACE demonstrated improved survival for the combination therapy for lesions > 3 cm without differences in terms of major complications (Lu et al., 2013; Chen et al., 2016). These improved outcomes may be explained in particular by the added antitumor efficacy *per se* of TACE on the targeted lesion, TACE-related decrease in heat dissipation due to tissue perfusion (hence decreasing the “heat-sink effect” responsible for decreased ablation efficacy) allowing RFA to achieve greater necrotic volumes, and the treatment of micrometastasis at the periphery of the ablated zone (Patterson et al., 1998; Lu et al., 2003). Interestingly, for lesions < 3 cm, no significant differences were found between the two treatment groups (Lu et al., 2013; Chen et al., 2016).

Another approach has been to combine RFA with adjuvant therapy. This combination therapy mainly aims at targeting the periphery of the ablated volume where sub-lethal temperatures are reached, with the rationale that increased cell cytotoxicity at the peripheral zone is likely to decrease local recurrence rate following ablation. Several combinatorial approaches of RFA with adjuvant nanoparticle-based chemotherapeutic agents (Solazzo et al., 2010; Yang et al., 2010, 2011, 2012; Moussa et al., 2014; Ahmed et al., 2015), chemotherapy (Hakime et al., 2007; Mertens et al., 2012; Ahmed et al., 2016) or radiation therapy (Horkan et al., 2005; Grieco et al., 2006) have been tested to maximize tumor destruction. The incorporation of a chemotherapeutic agent into a liposomal nanoparticle carrier has many mechanistic advantages such as a prolonged circulation time, increased tumor targeting, presumably through leaky tumor vessels, and decreased toxicity (Gabizon et al., 1994; Yuan et al., 1994; Ahmed M. et al., 2012). However, despite this favorable profile, intratumoral doses tend to be too low to achieve the desired antitumor effects, often being limited by systemic toxicities inherent to intravenous delivery. Therefore, further investigations are needed with optimized nanoparticle platforms and chemotherapeutical agents tailored to specific cancer types to improve antitumor efficacy targeting hyperthermia-related molecular pathways (Ahmed M. et al., 2012).

#### Novel Thermal Ablation Therapies

##### Irreversible electroporation

Irreversible electroporation (IRE) uses electric pulses to induce nanoscale defects in the cell membrane. These micro-perforations create homeostatic imbalances and subsequent cell death (Chang and Reese, 1990). In clinical practice, applicators are percutaneously inserted under imaging guidance (or *in situ* during surgery) into the tumors. Early evidence has shown that this ablative technique has the ability to preserve critical structures such as bile ducts, nerves and vessels (Rubinsky et al., 2007). Moreover, IRE raised hope of achieving better locoregional efficacy next to large vessels, when compared to other ablation techniques (RFA, MWA) susceptible to the heat-sink effect. Many cancer types, such as liver, lung, pancreas, prostate and kidney, have been treated with IRE. Gathered results in patients are summarized in a recent review (Wagstaff et al., 2016). Additionally, IRE seems particularly promising in the setting of combination therapy, with chemotherapy or nano-based platforms. Indeed, electroporation-induced cell membrane nanodefects not only lead to homeostatic impairment, but can also be used as channels to maximize cellular uptake of anticancer agents. In an orthotopic mouse model of pancreatic cancer, animals treated with the combination of IRE and systemic gemcitabine demonstrated significantly higher pancreatic drug concentration when compared to animals who received systemic gemcitabine only (Bhutiani et al., 2016). In the N1S1 rat model of hepatoma, animals received a systemic injection of SPIO nanoparticles loaded with doxorubicin (DOX-SPIO), followed by electroporation of the liver tumor lesions. Increased tumor uptake of theranostic nanoparticles was demonstrated on MRI (SPIO induces a marked decrease in the T2-weighted signal intensity of the tissues in which it accumulates) and histology (Mouli et al., 2013). Similarly, electroporation increased tumor uptake of iron oxide nanoparticles in a preclinical model of pancreatic cancer (West et al., 2014). Moreover, companion nanoreporter (^89^Zirconium-NRep) can be used for non-invasive quantitative PET monitoring of anticancer effects of electroporation and to predict treatment-mediated uptake of therapeutical nanoparticles (Srimathveeravalli et al., 2018).

##### High-intensity focused ultrasound

High-intensity focused ultrasound (HIFU) is another available ablative technology that uses an extracorporeal source of focused ultrasound energy and creates coagulation necrosis in a focal area without damaging normal surrounding tissue (Tempany et al., 2011). HIFU has shown promising results in several trials in different organs such as liver, kidney or brain (Kennedy et al., 2003). In liver disease, HIFU has been used for treatment of unresectable advanced stage HCC or for liver metastases. HIFU on HCC lesions has been shown to be safe, and to effectively improve the quality of life together with satisfactory tumor response (Ng et al., 2011). A retrospective analysis comparing TACE vs. TACE+HIFU showed better tumor response rates and median survival in the combined treatment group (Kim et al., 2012). Reduced dropout rates from liver transplantation waiting lists were obtained by HIFU, providing effective local tumor control (Cheung et al., 2013). Similarly, to e.g., electroporation, HIFU can be used in combination treatments, e.g., to increase intratumoral drug delivery. An interesting combined strategy was described in a pre-clinical model using temperature sensitive liposomes (TSLs) heated by non-invasive HIFU, leading to TSL increased permeability, local doxorubicin delivery, and in turn improved tumor response (Dromi et al., 2007). In addition, liposomal paramagnetic contrast agents have been reported, in which T1 relativity rapidly increases as temperatures rises, and can therefore be used for monitoring of MRI-guided thermal ablations (Frich et al., 2004; McDannold et al., 2004). A manuscript described the use of TSLs encapsulating both gadolinium-based contrast and doxorubicin, released upon ultrasound-induced hyperthermia and could demonstrated that the change in MR signal linearly correlated with the doxorubicin concentration in the tumor (de Smet et al., 2011). These findings need to be translated to clinical trials in human, but hold great promise since it means drug release could be probed *in situ* and potentially adapted in an almost real-time fashion.

##### Photothermal ablation

Another promising thermal ablation procedure is photothermal (PTT) ablation, which focuses a light source on a tumor and the absorbed energy is transformed into heat. This process can be optimized by tumor delivery/deposition of plasmonic photothermal sensitizers such as gold nanoparticles (O’Neal et al., 2004). Both sensitizers and laser light need to effectively reach the tumor in a specific and accurate manner. Laser radiation penetration is limited in tissues, which can be improved by bringing the laser in close vicinity to tumors through needles (Fang and Chen, 2013; Parchur et al., 2018). Most of the studies implicating PTT are still based on *in vitro* experiments or animal models. For example, a recent manuscript described a method of IR-guided photothermal therapy of colorectal cancer liver metastasis with theranostic gold nanorods (Parchur et al., 2018). In this work, optical/magnetic resonance and X-ray contrast bearing theranostic nanoparticles composed of gold were developed. After systemic administration, nanoparticles preferentially deposited in tumors because of the increased permeation and retention effect, but the delivered dose remained low. It was shown that this issue can be improved by site-specific delivery in the mesenteric vein of rats bearing colorectal liver metastasis, doubling the tumor-to-liver uptake as assessed by MRI, compared to systemic administration. After nanoparticle delivery image-guided PTT ablation was performed, and pathologic analysis of treated livers revealed thermal damage that was mostly confined to the tumor while sparing surrounding tissue (Parchur et al., 2018). Another study used gold nanoparticles (HAuNS) loaded with paclitaxel that were directly delivered in the hepatic artery followed by near-infrared laser irradiation (percutaneous photothermal therapy) of the tumors, achieving enhanced anticancer effects. Treatment efficacy resulted from high locoregional delivery of paclitaxel from the nanoembolization and the photothermal effect mediated by the nanoparticles themselves resulting not only in intratumoral heating but also triggering the release of the cytotoxic drug (Gupta et al., 2012). An important issue remains how to achieve specific and significant accumulation of nanoparticles in the tumors compared to adjacent tissue. This was addressed in another study which examined the effect of sequential intra-arterial administration of a vascular disrupting agent named combretastatin A-4 phosphate disodium (CA4P), followed by injection of doxorubicin-loaded, polyethylene glycol (PEG) coated hollow gold nanospheres (HAuNS) mixed with Lipiodol. It was shown that pre-treatment with CA4P induced a PEG-HAuNS–trapping effect in HCC that was subsequently exploited for PTT ablation, meanwhile triggering doxorubicin delivery, which in turn resulted in improved control of tumor growth compared to control (Li J. et al., 2016). The potential of nano-based delivery systems in PTT is promising given the numerous platforms available. However, the translation of this approach into the clinic will require further investigation to overcome limitations such biocompatibility, specific deposition in target tissue, heating efficiency and costs (Smith et al., 2015).

### Perspectives

In the past few decades, we significantly progressed in the molecular understanding of cancer. As a consequence, numerous targeted therapies were developed and nowadays constitute a cornerstone in the management of various malignancies. In parallel, image analysis has made tremendous progress. Some types of cancers such as HCC can be accurately diagnosed based on specific imaging parameters alone. Moreover, non-invasive, complex analysis of quantitative image features can sometimes be linked to tumor phenotypes. Indeed, extraction and analysis of data from medical images can provide information about a given oncologic tissue, e.g., some radiomic signatures can capture intra-tumoral heterogeneity and be linked to underlying gene-expression patterns (Aerts et al., 2014). In addition molecular imaging may provide real-time information of the whole tumor volume and its potential associated distant metastases. Artificial intelligence is also expected to play an increasing role in the quantitative analysis of images in the coming years. However, although research in the field of imaging is rapidly progressing, it remains of the utmost importance to obtain tumor tissue and paired blood-based samples not only at diagnosis but also in every treated site and in lesions evading treatment to identify novel molecular targets and predictors of treatment response. In-depth molecular analyses should account for inter-patient and intra-patient variability (different clonal evolution patterns may exist across tumors lesions within a patient at baseline and after treatment). Thus, the number of biopsies performed will significantly increase in the following years. Interventional oncologists will play a major role in performing a substantial amount of these biopsies using the most cutting-edge multimodal imaging techniques to precisely see, reach and target tumors lesions. Specific imaging probes, such as fluorescent probes and nanoplatforms will certainly play an important role in delineating healthy from malignant tissue and improving targeting. Paired imaging and tissue sampling analyses will likely lead to the development of new biomarkers of response, causing a paradigm shift in the assessment tumor response in the near future.

Interventional oncology is uniquely positioned to selectively reach tumor lesions not only for diagnostic purposes, but also for a wide range of minimally invasive percutaneous treatments such as thermal ablations that are increasingly being used for the treatment of HCC and other solid malignancies. The field of thermal ablations has evolved. Initially used as a way to completely eradicate small tumors, thermal ablations are increasingly being used in different clinical scenarios where they can be combined with other treatment modalities to maximize efficacy while reducing toxicity. Indeed, the ability of ablative technologies to precisely target tumor cells *in situ* by modifying the tumor microenvironment, cell permeability and tissue vascularization, and optimize drug delivery and uptake by targeted tissues, holds promise of achieving meaningful results for cancer patients. Ablations could thus be used in a neoadjuvant or adjuvant setting to maximize anticancer efficacy of systemic or locoregional chemotherapy, immunotherapy and/or theranostic nano-based therapeutics. Potential benefits for patients are multiple such as reduction in overall drug dose to reach treatment efficacy with subsequent reduced toxicity. Moreover, the use of nanotheranostics can provide additional valuable information about the treatment that is being delivered and be used as biomarkers for treatment prediction.

Refinements in the way TACE is performed allows to better identify tumor lesions and superselectively reach virtually any lesions in the liver. The advent of 3D imaging in the angiosuite will further evolve with new guidance tools and image fusion techniques. The use of theranostic molecules and nanoplatforms with imaging capabilities will likely develop to better identify, characterize and treat cancer lesions. The landscape of administered drugs is likely to change. TACE has been successfully performed using cytotoxic chemotherapeutic agents such as doxorubicin in combination with an embolic agent in HCC patients. However, TACE-related embolization is responsible for a cascade of detrimental effects such as the activation of proangiogenic factors in response to the hypoxic stress leading to poor outcomes. This established the clinical rationale to perform TACE using antiangiogenic drugs. This therapeutic strategy is actually under investigation and should be made available shortly for cancer patients (Bize et al., 2016; Duran et al., 2018). Similarly, targeting tumor hypoxia using hypoxia-activated prodrugs originally developed for systemic delivery were tested in locoregional delivery or in combination therapy (TACE + intravenous hypoxia-activated prodrug) in preclinical models for liver cancer and held promise in achieving meaningful results once translated in the clinic (Lin et al., 2016; Duran et al., 2017). The use of theranostic nanoparticules offers a myriad of possibilities in the setting of TACE where superselective disease targeting and high drug dose delivery can overcome challenges faced by systemic delivery such as biodistribution, tumor uptake and toxicity.

**FIGURE 8:**
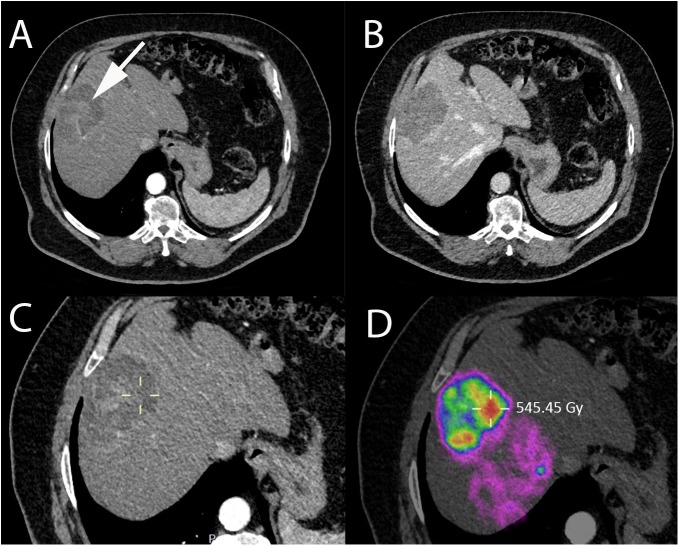
Immediate post-SIRT dosimetry using quantitative PET-CT with internal pair positron emission by ^90^Y in a 73-year-old patient with non-alcoholic steatohepatitis and HCC. **(A)** Contrast-enhanced CT-scan acquired during arterial phase showing HCC in segment VIII with heterogeneous areas of enhancement (white arrow). **(B)** Contrast-enhanced CT-scan acquired during portal phase showing contrast wash-out of the lesion. **(C)** Contrast-enhanced CT (arterial phase) and **(D)** quantitative PET-CT performed after SIRT with Y^90^-microspheres allowing direct assessment of the administered dose, expressed in Gray (Gy) using a region of interest (yellow cross).

Similarly, to TACE, efforts are undertaken to maximize tumor targeting during SIRT. Selectivity allows the administration of ever greater doses of radiation to the tumor while sparing the liver parenchyma. It is essential to achieve a certain tumor dose threshold to obtain an antitumoral effect, which can be anticipated with personalized dosimetric approaches and treatment intensification if needed (Garin et al., 2015). New theranostic solutions are needed to overcome discrepancies that can be observed between treatment planning and the treatment itself, to better predict the dose and optimize SIRT efficacy while minimizing toxicity. Substantial progress must be made in terms of dosimetry. A better understanding of the spatial distribution of the microparticules, the energy deposited in the tissues and the radiation field is needed (Walrand et al., 2014; Pasciak et al., 2016). In addition, a precise determination of the absorbed dose to the liver is fundamental for the safe development of this technique. Research should focus on immediate post-treatment dosimetry and improved image fusion techniques that can help identify undertreated regions and determine dose-effect and dose-toxicity relations. Image count-density maps of post-treatment ^90^Y PET-CT can already be converted into a dose-map, allowing direct assessment of the administered dose (Figure 8) and could be used e.g., for prediction of procedural success and outcomes. Moreover, the use of new-generation microparticules offers a theranostic solution for discrepancies between pretreatment scout dose imaging and the procedure itself with the added advantage of allowing for treatment monitoring during administration, and is likely to increase in the coming years. Selective intra-arterial administration of radiolabeled peptides is particularly interesting for the treatment of liver only or liver predominant cancer, with the ability of achieving a higher tumor absorbed radiation dose with decreased toxicity when compared to systemic delivery.

Many theranostic platforms for simultaneous cancer therapy and imaging applications are under development and include iron and manganese oxides, silica and gold nanoparticle-based systems (Ahmed N. et al., 2012). Iron oxide and manganese oxide nanoparticles can be combined with a variety of cytotoxic agents, monoclonal antibodies or siRNAs (Kohler et al., 2005; Lee et al., 2007, 2009; Bae et al., 2011), and because of their inherent superparamagnetic properties, can be used for drug delivery assessment using MRI. Similarly, luminescent silica nanospheres can be loaded with various agents and display increased imaging capabilities (Park J.H. et al., 2009). Gold nanoparticles can also serve as a platform to create multifunctional nanoprobes that sensitize tumoral tissues to radiation or photo-thermal therapy while increasing contrast for imaging (Ji et al., 2007; Huang et al., 2011). Carbon nanotubes can be employed to deliver a variety of therapeutic agents or perform gene therapy (Singh et al., 2005). Using navigation systems and multimodality imaging techniques to better identify and reach tumor lesions, these versatile nanoparticle-based theranostic systems can be intratumorally or locoregionally delivered, alone or in combination with systemic therapies, maximizing antitumor efficacy.

In conclusion, being at the crossroad of diagnosis and treatment, interventional oncology is uniquely positioned to play a growing role in personalized care of cancer patients. Many theranostic systems are currently under development and will help interventional oncologists achieve ever more precise and accurate image-guided minimally invasive endo-vascular and percutaneous procedures that are tailored to the patient characteristics.

## Author Contributions

All authors wrote the manuscript. ND and RD assembled and designed the figures.

## Conflict of Interest Statement

The authors declare that the research was conducted in the absence of any commercial or financial relationships that could be construed as a potential conflict of interest.

## Abbreviations

^166^Ho: holmium-166
^177^Lu-DOTATATE: [lutetium-177 DOTA^0^,Tyr^3^]octreotate
^90^Y: yttrium-90
^90^Y-DOTATOC: [^90^Y-DOTA^0^ Tyr^3^]octreotide
^99m^Tc-MAA: technetium-99m-macroaggregated albumin
Ad-MAA: adamantine functionalized MAA
BCLC: Barcelona Clinic Liver Classification
CBCT: cone-beam computed tomography
cTACE: conventional TACE
DEB-TACE: drug-eluting beads TACE
DSA: digital subtraction angiography
GEP-NETs: gastroenteropancreatic neuroendocrine tumors
HCC: hepatocellular carcinoma
HIFU: high-intensity focused ultrasound
ICG: indocyanine green
IRE: irreversible electroporation
MRI: magnetic resonance imaging
MWA: microwave ablation
PET-CT: positron emission tomography–computed tomography
PTT: photothermal
RFA: radiofrequency ablation
SIRT: selective internal radiation therapy
SPIO: superparamagnetic iron oxide
TACE: transarterial chemoembolization
TSLs: temperature sensitive liposomes
